# Diet, Exercise, and Lifestyle in Glaucoma: Current Evidence and Future Perspectives

**DOI:** 10.3390/nu17213369

**Published:** 2025-10-27

**Authors:** Akiko Hanyuda, Satoru Tsuda, Noriko Himori, Kota Sato, Naoki Takahashi, Toru Nakazawa

**Affiliations:** 1 Department of Ophthalmology, Keio University School of Medicine, Shinanomachi, Shinjuku-ku, Tokyo 160-8582, Japan; akihanyu@keio.jp; 2 Department of Ophthalmology, Tohoku University Graduate School of Medicine, Seiryo-machi, Aoba-ku, Sendai 980-8574, Miyagi, Japan; 3 Epidemiology and Prevention Group, Center for Public Health Sciences, National Cancer Center, Tokyo 104-0045, Japan; 4 Division of Ophthalmic Precision Medicine Development, United Centers for Advanced Research and Translational Medicine (ART), Tohoku University Graduate School of Medicine, Seiryo-machi, Aoba-ku, Sendai 980-8575, Miyagi, Japan; 5 Department of Aging Vision Healthcare, Tohoku University Graduate School of Biomedical Engineering, Aramaki Aza Aoba, Aoba-ku, Sendai 980-8579, Miyagi, Japan; 6 Department of Advanced Ophthalmic Medicine, Tohoku University Graduate School of Medicine, Seiryo-machi, Aoba-ku, Sendai 980-8574, Miyagi, Japan; 7 Department of Retinal Disease Control, Tohoku University Graduate School of Medicine, Seiryo-machi, Aoba-ku, Sendai 980-8574, Miyagi, Japan

**Keywords:** diets, exercise, glaucoma, lifestyle, sleep, public health

## Abstract

Glaucoma is a major ocular neurodegenerative disease and a leading cause of irreversible blindness worldwide, with prevalence projected to exceed 110 million by 2040. Although lowering intraocular pressure (IOP) remains the only proven treatment, glaucoma arises from a complex interplay of genetic, local, and systemic factors—including oxidative stress, vascular dysregulation, mitochondrial dysfunction, and neuroinflammation. Emerging evidence suggests that modifiable lifestyle factors may influence these pathogenic pathways. In this review, higher dietary nitrate from leafy greens is consistently associated with lower primary open-angle glaucoma risk, aligning with nitric-oxide-mediated endothelial support and more stable ocular perfusion pressure. Flavonoids (anthocyanins and flavanols), carotenoids (lutein/zeaxanthin), and B vitamins have strong biological rationale for glaucoma prevention but have limited support from long-term, large population-based studies. The effect of polyunsaturated fats on glaucoma remains inconsistent and warrants source-(plant vs. animal) and substitution-based analyses. Consistent protective effects of aerobic exercise and high-quality sleep may be associated with favorable metabolic profiles and ocular perfusion, potentially mitigating retinal ganglion cell loss. Conversely, smoking and alcohol use are frequently coupled with poorer diet quality (e.g., lower vegetable intake) and heightened oxidative stress, which may exacerbate glaucomatous neurodegeneration. However, much of the current literature is constrained by cross-sectional designs, reliance on self-reported food frequency questionnaires, and insufficient use of structural endpoints such as retinal nerve fiber layer imaging. This review focuses on the potential of lifestyle modification and future directions in prevention and treatment strategies for glaucoma, highlighting the need for large-scale, multi-ethnic, genotype-stratified longitudinal studies and randomized controlled trials to establish causality and define optimal intervention strategies.

## 1. Introduction

Glaucoma is a progressive neurodegenerative optic neuropathy and one of the leading causes of irreversible blindness worldwide, currently affecting an estimated 76 million individuals—a figure expected to rise substantially with global population aging [1]. The disease is characterized by the degeneration of retinal ganglion cells (RGCs) and their axons, leading to characteristic visual field defects and permanent vision loss [2]. Although lowering intraocular pressure (IOP) is the only proven prevention and therapy [3], multiple interrelated mechanisms, including vascular dysregulation, oxidative stress, immune-inflammatory activation, excitotoxicity, and mitochondrial dysfunction, are implicated in RGC injury [4,5].

Growing evidence suggests that lifestyle factors—particularly dietary patterns and specific nutrients, as well as exercise, smoking, sleep, alcohol, and caffeine—may modulate underlying pathophysiological mechanisms of glaucoma [6,7,8]. For instance, dietary anti-antioxidants (e.g., carotenoids, vitamins C and E, and polyphenols) and one-carbon-pathway nutrients (e.g., folate and vitamins B-2/B-6/B-12) support glutathione regeneration and homocysteine control, thereby mitigating oxidative and nitrosative stress implicated in RGC injury [9,10,11]. Conversely, smoking increases oxidant burden and depletes antioxidant and B-vitamin pools, amplifying lipid peroxidation and protein/DNA damage [12]; zinc deficits may further impair antioxidant enzymes and taste-guided food choices [13,14,15]. Omega-3 fatty acids and anti-inflammatory dietary patterns can dampen microglial activation and neuroinflammatory cascades [16,17], whereas excess refined carbohydrates and adverse adipokine signaling may have the opposite effect [18].

Vascular mechanisms are suggested to be nutrition-responsive. Dietary nitrate (leafy greens) augments nitric oxide (NO) bioavailability and endothelial function, potentially stabilizing ocular perfusion pressure and microvascular flow at the optic nerve head [19]. Exercise improves endothelial function, insulin sensitivity, and mitochondrial biogenesis, and acutely lowers IOP in many contexts [20,21]—effects that may be potentiated by adequate nutrient status (e.g., antioxidant sufficiency, omega-3 availability). In contrast, smoking impairs endothelial NO signaling and promotes vasospasm [12], while sleep disorders (e.g., obstructive sleep apnea [OSA]) drive intermittent hypoxia and oxidative stress [22] and can exacerbate nocturnal hypotension, a recognized vascular stressor for glaucoma [23]. If these exposures can be harnessed to favorably shift those mechanisms, they may complement IOP-lowering strategies to reduce disease risk and slow progression.

Prior reviews typically catalog exposures (e.g., “antioxidants,” “exercise”) or focus on single factors without explaining how they modulate glaucoma mechanisms and rarely explore the mechanistic insights on the relation between various lifestyle factors and glaucoma. Herein, guided by this mechanism-first framework, the present narrative review synthesizes evidence on how lifestyle and dietary factors influence the incidence and progression of glaucoma and outlines future directions for targeting modifiable risks to complement conventional therapeutic strategies. We searched PubMed (MEDLINE) for studies published prior to 30 September 2025. The Medical Subject Headings (MeSH) and keywords included “glaucoma/diet therapy” or (“glaucoma” and (“physical activity” or “exercise”) or “lifestyle” or (“smoking” or “cigarette”) or (“sleep” or “sleep apnea” or “sleeping behaviors”) or “diet, food, and nutrition” or “feeding behavior” or “nutrition therapy” or “trace element” or “minerals” or “carotenoids” or “flavonoids” or “fatty acids”) or (“alcohol” or “drinking habits”) or (“coffee consumption” or “caffeine”). Unless otherwise specified, we limited inclusion to English-language human epidemiological or clinical studies with an available title and abstract and prioritized randomized controlled trials, systematic reviews, and meta-analyses. After initial screening by title and abstract, we manually reviewed all the full texts of eligible records and further examined their reference lists to include relevant literature on the area of interest. Observational clinical studies in this review were required as follows: (i) studies evaluated the association between lifestyle factors and prevalence/risk of glaucoma, (ii) subjects were adults with confirmed presence of glaucoma, and (iii) peer-reviewed articles.

## 2. Exercise

Accumulating evidence indicates that regular aerobic exercise may confer both IOP-lowering and neuroprotective benefits in glaucoma [20,21,24]. In an experimental model of glaucoma induced by elevated IOP, aged mice that engaged in voluntary exercise maintained brain-derived neurotrophic factor (BDNF) levels comparable to those of young sedentary mice and exhibited similar preservation of RGCs [20]. These findings suggest that exercise may protect RGCs through mechanisms such as increased dopamine and neurotrophic factor expression and enhanced systemic and ocular blood flow.

In humans, a prospective cohort study using accelerometers to objectively measure activity demonstrated that walking 5000 steps per day or reducing sedentary time by 2.6 h daily was associated with an approximately 10% reduction in the rate of visual field progression [24]. Similarly, an independent predictor of a slower progression rate of MD slope was reported in a longitudinal study with a median follow-up of 4.9 years in patients with primary open-angle glaucoma (POAG) [25]. Meta-analytic data also support a significant IOP-lowering effect of exercise—despite heterogeneity between studies—with the most pronounced benefit observed in sedentary individuals [21].

However, certain activities can transiently elevate IOP and may be detrimental in specific patient groups. In pigment dispersion glaucoma, vigorous exercise can trigger IOP spikes. Head-down yoga postures, such as Sirsasana, may induce doubled IOP within minutes, and case reports have linked such positions to visual field deterioration [26]. Other inverted yoga poses, activities involving abrupt head movements, and playing wind instruments have shown similar transient effects [27]. Swimming with tight-fitting goggles can also cause brief IOP elevations; although large epidemiological studies have not confirmed long-term structural damage, caution—especially after filtering surgery—is warranted [28,29].

Overall, being physically fit and maintaining regular aerobic activity appears protective against glaucoma onset, with dose–response evidence in male runners showing lower incidence with greater running distance and speed [30]. Nonetheless, large cohort data indicate that extreme exercise was related to adverse effects of glaucoma [31], and a review suggested an adverse association between very vigorous daily exercise and glaucoma [32], possibly due to the strenuous exercise-induced hypoxia [33]. A recent interventional study showed a greater transient increase in ocular perfusion pressure after walking at high speed than at slow speed among POAG [34]. These lines of evidence suggest that moderate exercise can have a protective effect against glaucoma [35].

Beyond its role in reducing IOP and promoting neuroprotection, exercise exerts systemic effects that interact with nutritional status and metabolism, both of which are directly relevant to ocular health. For example, aerobic exercise has been shown to improve insulin sensitivity and lipid metabolism, thereby influencing nutrient availability and utilization [36,37]. Improved circulation and mitochondrial efficiency following regular exercise may enhance the delivery and metabolism of micronutrients such as antioxidants (e.g., vitamins C and E) [9,10,11] and omega-3 fatty acids [16,17], which are critical for retinal and optic nerve protection. Moreover, exercise-induced upregulation of BDNF [38] and NO signaling may be potentiated by adequate nutritional status, suggesting a synergistic interaction between diet and physical activity in maintaining optic nerve resilience [19]. Thus, considering exercise and nutrition together may provide a more effective strategy for glaucoma risk modification.

## 3. Smoking

Tobacco smoking is a well-recognized risk factor for systemic vascular disorders, including atherosclerosis and ischemic heart disease, and is known to impair microcirculatory function [12]. Although nicotine—the principal active compound in tobacco—can increase systemic blood flow, its influence on optic nerve head perfusion remains uncertain. Epidemiological investigations examining the relationship between cigarette smoking and POAG have yielded inconsistent results. A large prospective cohort study involving more than 100,000 participants [39] and a recent systematic review and meta-analysis [40] reported no significant association between smoking and POAG incidence, while a case–control analysis suggested a potential protective association with NTG [41]. Conversely, other population-based studies and a meta-analysis identified smoking as a risk factor for glaucoma development [42,43,44]. These discrepancies may reflect the complex pharmacological profile of nicotine, which has been shown in experimental models to exert neuroprotective effects—such as attenuation of glutamate-induced excitotoxicity in RGCs—yet also exhibits vasoconstrictive properties that may reduce optic nerve blood flow.

Although evidence has been limited, heavy smokers are likely to exhibit poorer nutrient profiles [14,15]. In a Japanese cohort, cigarette smokers had lower protein–energy and fat–energy ratios and lower intakes of certain vitamins (vitamins B1, B2, C, and K), minerals, and fiber and consumed more alcoholic drinks than non-smokers [45], potentially reflecting altered appetite and food preferences (e.g., nicotine-related appetite suppression, taste effects of propylene glycol/glycerol carriers, and lower zinc intake that may blunt taste) [14,15]. Taken together, smoking appears linked to lower antioxidant and micronutrient availability (e.g., vitamins C and B-complex and zinc), a pattern biologically poised to exacerbate oxidative stress and endothelial dysfunction, two pathways central to glaucomatous optic neuropathy [5,10]. Mechanistically, reduced antioxidant/NO bioavailability and one-carbon metabolism disturbances (e.g., folate/B-vitamin insufficiency with homocysteine accumulation) may amplify reactive oxygen species, impair microvascular regulation (including optic nerve head and choroidal perfusion), and accelerate structural/functional loss. Observational imaging studies further associate smoking intensity with lower optic nerve head vessel density and greater choroidal microvasculature dropout, supporting a vascular mechanism [46,47,48]. Thus, beyond direct toxic and hemodynamic effects, smoking-related nutrient deficits likely act as modifiable amplifiers of oxidative and vascular injury in glaucoma.

## 4. Sleep

Sleep occupies approximately one-third of human life and is a fundamental physiological process. Increasing evidence indicates that insufficient or poor-quality sleep is associated with various chronic conditions, including diabetes, obesity, and increased mortality [22]. In recent years, attention has also turned to the relationship between sleep and glaucoma, with a particular focus on OSA as a potential risk factor [49].

A 5-year longitudinal study, matching participants with OSA to controls by age and sex, reported a hazard ratio of 1.67 for glaucoma in the OSA group [23]. Initially, it was hypothesized that nocturnal apneic episodes might trigger glaucoma by elevating IOP through increased intrathoracic pressure. However, subsequent investigations revealed that IOP may actually decrease during apnea events [50]. While the precise mechanism remains unclear, ischemia and hypoxia induced by OSA are considered likely contributors to glaucomatous optic nerve damage. Supporting this hypothesis, a study in Japanese glaucoma patients found that those with OSA exhibited significantly higher systemic oxidative stress levels and faster mean deviation slope deterioration compared with non-OSA patients [51]. Furthermore, in patients with broad-spectrum POAG and comorbid OSA, the introduction of continuous positive airway pressure therapy led to reductions in oxidative stress markers and a deceleration of visual field progression [52].

Regarding sleep duration and glaucoma, a growing body of evidence suggests a non-linear, U-shaped association in large-scale cohort studies [53,54]. Using optical coherence tomography (OCT), sleep duration of <6 h was independently associated with a thinner retinal nerve fiber layer (RNFL), and RNFL was thickest among those who slept for 6–7 h [54].

Collectively, these findings highlight the importance of adequate, high-quality sleep as part of both glaucoma prevention and management. In particular, early detection and treatment of OSA may offer therapeutic benefits in mitigating glaucomatous progression.

## 5. Nutrient and Diet

In this section, we have divided major energy sources (carbohydrates, proteins, and fats) and other nutrients. Major population-based studies with large sample sizes (*n* > 500) cited in this review are summarized in Table 1.

### 5.1. Major Energy Sources

#### 5.1.1. Carbohydrates

Prospective evidence on carbohydrate intake and glaucoma risk is limited and inconsistent [55,56]. In one cohort study, participants in the highest quartile of carbohydrate consumption at baseline had a significantly greater risk of glaucoma compared with those in the lowest quartile (hazard ratio [HR] 1.50; 95% confidence interval [CI], 1.01–2.25; Ptrend = 0.042), independent of diabetic status [56]. However, another large cohort found no significant associations between three types of low-carbohydrate diet scores (overall, animal-based, and vegetable-based) and overall POAG risk [55]. Notably, the vegetable-based scores showed a non-significant trend toward an inverse association with the POAG subtype presenting with early paracentral visual field loss (highest vs. lowest decile multivariable-adjusted relative risk [MVRR] 0.78; 95% CI, 0.55–1.10; Ptrend = 0.12) [55], suggesting that substituting vegetable-derived fats and proteins for carbohydrates may reduce risk for this specific disease phenotype.

#### 5.1.2. Proteins

Several observational studies have examined animal protein intake in relation to glaucoma. In a Japanese cross-sectional study, higher meat consumption frequency was inversely associated with OAG in women (odds ratio [OR] 0.61; 95% CI, 0.43–0.88) [57]. Similar findings from a Greek study suggested that POAG patients consumed less meat than controls, prompting the authors to recommend adequate meat intake for individuals at risk [58].

Regarding specific amino acids, a large prospective analysis from the Nurses’ Health Study and Health Professionals Follow-Up Study investigated dietary branched-chain amino acids (BCAA: leucine, isoleucine, valine) in relation to POAG [59]. Across nearly 1950 incident POAG cases, no significant association was found between high BCAA intake and overall POAG risk (MVRR 0.93; 95% CI, 0.73–1.19), though a modest, non-significant inverse trend was noted in women with early paracentral visual field loss.

Fish-derived protein also appears relevant: in a prospective cohort and nested case–control analysis, greater adherence to the Mediterranean–DASH Intervention for Neurodegenerative Delay (MIND) diet, which includes regular fish consumption, was associated with a lower risk of POAG (OR 0.80; 95% CI, 0.66–0.96 for each 10% increase in adherence) [60].

#### 5.1.3. Fats

Findings on dietary fat and glaucoma risk are heterogeneous. Two prospective cohort studies reported a positive association between a high dietary omega-3 to omega-6 fatty acid ratio and increased glaucoma risk (RR 1.49; 95% CI, 1.11–2.01 and HR 1.91; 95% CI, 1.05–3.46) [61,62], with the adverse association particularly evident in high-tension POAG [61], whereas no significant associations were observed for omega-3 or omega-6 intake individually. In contrast, a cross-sectional study of Japanese Americans found that lower vegetable fat intake was associated with higher glaucoma prevalence [63], suggesting a potential protective role for plant-based fats. Conversely, another cross-sectional study reported that glaucoma patients had higher visible fat intake compared with non-glaucoma controls and suggested reducing total fat consumption as prudent advice for at-risk individuals [58]. A recent finding from the UK Biobank cohort suggested the protective effect of plasma levels of omega-3 fatty acids on POAG was stronger in those with high genetic risk scores of POAG (although this association was not statistically significant), and this causal association was confirmed by a Mendelian randomization study [64]. These discrepancies likely reflect several factors: (i) fat sources and substitution patterns (e.g., plant-derived unsaturated fats vs. animal fats), (ii) exposure metrics (omega-3 to omega-6 ratio vs. absolute intakes), and (iii) disease subtypes (high-tension vs. normal-tension POAG). Accordingly, source-specific and substitution-aware analyses, ideally supported by biomarkers of fatty acid status (e.g., plasma phospholipids) and dietary-pattern approaches, are needed to clarify whether plant-based unsaturated fats are protective and whether an elevated omega-3 to omega-6 ratio in some settings merely proxies other dietary features rather than a direct causal effect.

### 5.2. Vitamins and Antioxidant Properties

#### 5.2.1. Vitamins and Provitamins

A number of epidemiological studies have explored the association between dietary intake of vitamins and the risk of glaucoma, though findings remain inconsistent. Cross-sectional data in older African American women demonstrated inverse associations between glaucoma prevalence and high intakes of vitamin A, vitamin C, and α-carotene, with borderline trends for β-carotene, folate, and lutein/zeaxanthin [65]. Another study reported that low vitamin A intake was associated with higher glaucoma risk (OR 0.37; *p* = 0.019) [63].

Using large population-based US cohorts, Kang JH and her colleagues prospectively suggested possible protective roles for certain B vitamins [66]. Higher folate (vitamin B9) intake was associated with a lower risk of exfoliation glaucoma (XFG) or glaucoma suspect status (highest vs. lowest quintile MVRR 0.75; Ptrend = 0.02). Similarly, higher intake of retinol equivalents and vitamin B1 was associated with reduced OAG risk (HR 0.45 and 0.50, respectively) [67]. In contrast, a cross-sectional analysis linked high niacin (vitamin B3) intake to increased glaucoma risk [68]. Several large cohorts have found no significant associations between intake of vitamins A, C, E, D, or carotenoids and glaucoma incidence, including studies using dietary data, supplement use, and serum levels [69,70].

#### 5.2.2. Nitric Oxide

NO, derived from dietary nitrate (notably from dark green leafy vegetables) and endothelial production, enhances ocular blood flow [81]. In glaucoma, it is thought that autoregulatory function is disrupted due to factors such as elevated IOP, endothelial dysfunction, and activation of astrocytes [82]. Vascular endothelial cells possess barrier functions, maintain vascular homeostasis, and produce vascular-tone-regulating factors such as NO and endothelin-1 (ET-1) [83]. NO relaxes vascular smooth muscle and induces vasodilation, but when NO production is inhibited, vasospasm of the short posterior ciliary arteries has been reported. Indeed, glaucoma patients have been found to exhibit reduced NO levels and compromised ocular hemodynamics [84,85]. In a clinical study, topical nipradilol increased the blood velocity of the optic nerve head in humans [86]. Dietary studies show that high consumption of nitrate-rich vegetables is associated with lower POAG risk, particularly for subtypes with early paracentral visual field loss (e.g., MVRR 0.52 for highest vs. lowest intake) [71,72]. However, it remains uncertain whether this benefit is attributable to nitrate content specifically or to coexisting antioxidant compounds.

#### 5.2.3. Carotenoids

Carotenoids include xanthophylls (e.g., lutein and zeaxanthin) and carotenes (e.g., α-carotene and β-carotene). Their primary dietary sources include leafy greens, carrots, and other pigmented vegetables and fruits. The xanthophyll carotenoids lutein and zeaxanthin accumulate in the macula as macular pigment, where they filter short-wavelength light and act as antioxidants [87]. Experimental and translational data indicate additional effects on redox homeostasis and neuroinflammation relevant to RGC health [88].

Earlier evidence linking carotenoids to glaucoma risk is inconsistent [65,67,69,71]. Prospective cohort analyses that treated carotenoids within broad “antioxidant” exposures have generally shown null associations with incident POAG (e.g., Nurses’ Health Study/Health Professionals Follow-Up Study), underscoring the need for more targeted carotenoid metrics [69], although one study reported a significant protective effect of high α-carotene intake but not for other carotenoids [65]. Recently, studies that directly assessed the macular pigment optical density (MPOD, a tissue proxy for lutein/zeaxanthin status) have increased [89,90]. In the Carotenoids in Age-Related Eye Disease Study 2 (CAREDS2), lower MPOD was associated with thinner macular retinal layers, including the ganglion cell complex, in eyes with and without manifest POAG [90]. Collectively, although carotenoid-rich dietary patterns remain biologically plausible, population-based evidence is still limited for POAG.

#### 5.2.4. Flavonoids

Flavonoids (e.g., anthocyanins and flavanols) are abundant in berries, tea, cocoa, and certain plant extracts. Flavonoids directly scavenge reactive oxygen species and upregulate the Nrf2/ARE pathway, increasing phase II enzymes (HO-1, NQO1) and glutathione synthesis—responses that are neuroprotective in RGC injury models [91,92]. In addition, flavonoids may enhance ocular blood flow, improve endothelial function, and confer neuroprotection independent of IOP lowering [73,74,75]. Indeed, supplementation with blackcurrant anthocyanins has been shown in randomized trials to reduce IOP and improve visual fields in POAG patients, with associated reductions in endothelin-1 levels [73]. Dark chocolate flavonoids have been shown to induce retinal vasodilation in healthy individuals, though not in glaucoma patients, potentially due to preexisting endothelial dysfunction [76]. In an 8-week clinical trial, supplementation with plant-based flavonoids (e.g., hesperidin, crocetin, and Tamarindus indica) significantly reduced plasma oxidative stress levels among those with high oxidative stress levels but not among those with low oxidative stress levels [77]. A meta-analysis reported beneficial effects of flavonoids on visual field indices in glaucoma but no effect on IOP [93]; however, findings were driven largely by one large trial [78], and long-term population-based data (e.g., Nurses’ Health Study) did not confirm a protective association [79].

#### 5.2.5. Fruits, Vegetables, and Composite Dietary Patterns

Multiple studies indicate that higher consumption of fruits and vegetables, particularly those rich in antioxidants, is associated with reduced glaucoma risk [65,71,72]. Cross-sectional studies report substantially lower odds of glaucoma in individuals with frequent intake of leafy greens (collards, kale), carrots, peaches, and oranges [71]. For example, ≥1 serving/month of collards or kale was associated with a 69% lower glaucoma risk, while ≥2 servings/week of carrots conferred a 64% reduction. Similarly, in African American women, ≥3 servings/day of fruit or juice was associated with a 79% lower risk [65].

Prospective evidence aligns with these findings: higher intake of green leafy vegetables was associated with lower POAG risk overall (MVRR 0.82 for the highest vs. lowest quintile) and particularly for POAG with early paracentral field loss (MVRR 0.52) [72]. Berries and leafy greens also appeared protective in nested case–control studies [60]. By contrast, a randomized dietary intervention increasing fruit, vegetable, and grain intake while reducing fat did not alter POAG incidence over follow-up [80]. Fruits may exert both protective effects, via vitamins and polyphenols, and adverse effects, through oxidative stress induced by fructose; thus, their net impact may vary depending on the underlying pathogenic mechanism of glaucoma. Future research should therefore prioritize comprehensive dietary patterns rather than focusing solely on individual nutrients or food groups.

### 5.3. Minerals

#### 5.3.1. Calcium, Iron, and Magnesium

Both calcium and iron participate in oxidative stress pathways and have been linked to glaucoma risk [94,95]. Large population-based analyses (National Health and Nutrition Examination Survey [NHANES]) reported that high supplemental calcium and iron intake was associated with increased odds of self-reported glaucoma (calcium: OR 2.44; iron: OR 3.80 for highest vs. lowest quintile), whereas higher dietary intake of these minerals was paradoxically associated with lower glaucoma odds [95]. This discrepancy may be explained by differences in bioavailability, absorption kinetics, and peak plasma levels between dietary and supplemental sources.

Mechanistically, excess iron catalyzes the formation of reactive oxygen species via the Fenton reaction, potentially damaging trabecular meshwork cells and impairing aqueous humor outflow [96]. High serum ferritin levels have been positively associated with glaucoma risk in multiple cohorts [94]. Conversely, in women with iron-deficiency anemia, thinner peripapillary RNFL has been reported, although causality remains unclear [97]. Calcium, in turn, is involved in intracellular signaling in both the trabecular meshwork and RGCs, with impaired regulation implicated in neurodegeneration [98]. While some observational studies have found no association between dietary calcium and OAG [67], the possibility of a detrimental effect from high-dose supplements warrants further investigation.

High dietary magnesium intake has been linked to an increased OAG risk (highest tertile OR 2.25 in the Rotterdam Study) [67]. Given magnesium’s calcium-channel-blocking properties and its role in vascular smooth muscle relaxation, excessive intake might theoretically exacerbate ocular hypoperfusion in susceptible individuals.

#### 5.3.2. Selenium and Other Trace Elements

Selenium, an essential component of glutathione peroxidase, plays a key role in antioxidant defense [99]. Findings are conflicting: one case–control study reported higher plasma selenium levels in POAG (OR 11.3 for the highest vs. lowest tertile), while aqueous humor selenium levels in the middle tertile were associated with the lowest glaucoma odds [100]. Experimental data suggest that excess selenium can increase outflow resistance in trabecular meshwork cells [101].

Other trace elements—such as zinc, copper, manganese, and mercury—have also been studied. Elevated serum manganese, molybdenum, and mercury have been reported in exfoliation syndrome (XFS) and XFG [102]. In the Korean NHANES, blood manganese showed an inverse association with glaucoma, whereas blood mercury showed a positive association [103]. No significant associations were found for arsenic or cadmium [67].

## 6. Caffeine

Caffeine (1,3,7-trimethylxanthine) is a methylxanthine alkaloid found in many widely consumed beverages [104]; a single cup of coffee or green tea contains approximately 100 mg of caffeine. Its primary pharmacological actions—adenosine receptor antagonism and phosphodiesterase inhibition—elevate intracellular cyclic AMP levels, which can influence aqueous humor dynamics by (1) increasing aqueous production, (2) reducing trabecular meshwork smooth muscle tone and closing fenestrations, and (3) raising systemic blood pressure, thereby increasing hydrostatic pressure for aqueous formation [105].

Population-based and prospective cohort studies have generally found no significant association between total caffeine consumption and POAG prevalence/incidence [106,107,108]. However, secondary analyses suggest that in individuals with a family history of glaucoma, higher caffeine intake may increase the risk of high-tension POAG. However, findings from large prospective cohorts, such as a U.S. longitudinal study, suggest that high consumption—≥5 cups per day—may significantly increase glaucoma risk (Ptrend = 0.02) [106]. Similarly, UK Biobank data indicate that in individuals with a high polygenic risk score for glaucoma, heavy coffee consumption may sharply elevate disease risk [107]. Additional risk stratification analyses are required to further elucidate the effect of caffeine on glaucoma.

Caffeine consumption has also been linked to an increased risk of XFG but not NTG [109]. This may be mechanistically plausible, as coffee intake elevates plasma and aqueous humor homocysteine levels, and hyperhomocysteinemia has been implicated in the pathogenesis of XFS/XFG [110,111]. In the Nurses’ Health Study and Health Professionals Follow-up Study, high coffee consumption was associated with a 63% increased risk of XFG, whereas dietary factors that lower homocysteine appeared protective, underscoring a possible metabolic link between caffeine, homocysteine metabolism, and XFS pathophysiology [109].

## 7. Alcohol Intake

Alcohol consumption has long been investigated as a potential modifiable risk factor for glaucoma, but epidemiologic findings remain inconsistent. Meta-analytic evidence suggests that drinkers have approximately a 22% higher incidence of POAG compared with non-drinkers [112], although large prospective cohorts have yielded conflicting results—ranging from increased risk at ≥7 drinks per week in the Black Women’s Health Study [43] to a non-significant protective association in the Nurses’ Health Study and Health Professionals Follow-up Study [113].

Population-specific differences in race, sex, and lifestyle may partly explain these discrepancies. Proposed mechanisms for alcohol-related glaucomatous damage include oxidative stress, sympathetic activation with transient IOP elevation, vitamin B1 deficiency, direct neurotoxicity, and indirect vascular impairment. Recent large-scale imaging studies, including the UK Biobank [114], Gutenberg Health Study [115], and Beaver Dam Offspring Study [116] have demonstrated dose-dependent thinning of the RNFL and macular ganglion cell layer (GCL) in association with alcohol intake, with threshold effects observed at intakes as low as ~50 g/week—below current UK [117] and US alcohol guidelines [118]. Mendelian randomization analyses further support a potential causal link between alcohol consumption and macular GCL loss [114].

For XFS/XFG, evidence is scarce but noteworthy. While Asian [119,120] and Icelandic [121] studies report no association or even a protective effect of moderate drinking, pooled analyses from three large US cohorts indicate a dose-dependent increase in XFG risk, with beer, wine, and whiskey consumption all contributing, except for a non-significant protective trend with red wine [122]. Mechanistically, alcohol may promote XFG pathogenesis through folate depletion and impaired homocysteine metabolism, whereas the polyphenol-rich antioxidant content of red wine may confer partial neuroprotection. Collectively, the literature suggests that high alcohol intake is more often associated with adverse structural and functional outcomes in glaucoma, and moderation—particularly in individuals with elevated genetic or clinical susceptibility—may represent a prudent preventive strategy.

## 8. Limitations and Future Perspectives for Lifestyle Modifications in Glaucoma Management

Glaucoma is a heterogeneous disease, and its risk factors include intrinsic and extrinsic factors, including genetic [123,124,125], oxidative stress [126], vascular dysregulation [127], and metabolic factors [128]. In the future, assessing patient predisposition using polygenic risk scores as well as measuring oxidative stress and ocular blood flow may help identify at-risk individuals, enabling lifestyle modifications aimed at mitigating adverse conditions and thereby contributing to glaucoma prevention.

**Figure 1:**
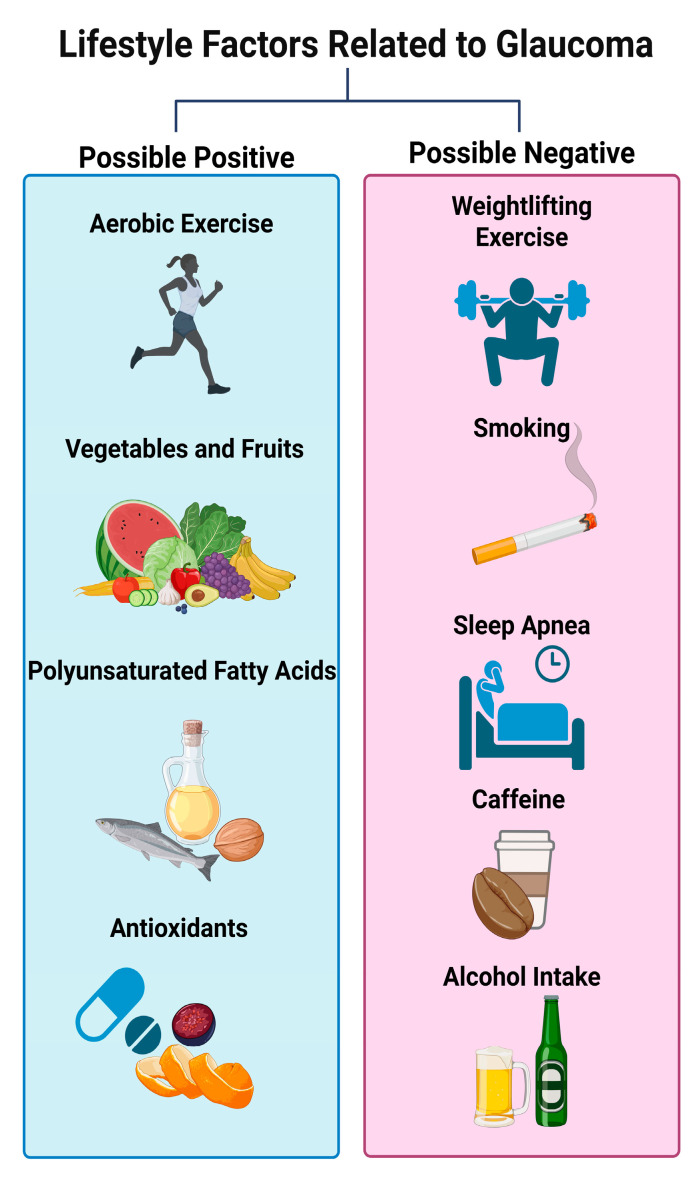
Lifestyle modifications related to glaucoma discussed in this review (created in BioRender. Hanyuda, A. (2025). https://BioRender.com/9m2clal (accessed on 24 October 2025)).

In this review, modifiable lifestyle factors—including balanced dietary habits, regular physical activity, adequate sleep quality, smoking cessation, and moderation of caffeine and alcohol intake—may beneficially influence glaucoma-related pathogenic mechanisms such as oxidative stress, vascular dysregulation, mitochondrial dysfunction, and neuroinflammation (Figure 1).

Nonetheless, while these associations are biologically plausible and supported by experimental and epidemiological data, the current body of evidence remains insufficient to warrant strong clinical recommendations. Many existing studies are limited by uncontrolled designs, potential regression-to-the-mean effects for IOP measurements, learning effects in subjective visual function testing, and the absence of objective structural endpoints such as OCT-derived RNFL or GCL assessments.

To advance the field, future research should prioritize well-designed longitudinal studies in diverse, multi-ethnic, and genotype-stratified populations, as well as rigorously conducted randomized controlled trials, to establish causal relationships and define optimal thresholds for beneficial or harmful exposures. Importantly, lifestyle interventions should be regarded as complementary to, rather than substitutes for, established IOP-lowering therapies.

## 9. Conclusions

Although the current review suggests that lifestyle may interact with nutrition, thereby influencing eye health, most studies are cross-sectional with a short follow-up period, and evidence from randomized controlled trials is lacking. At this stage, it is difficult to advise patients with glaucoma on modifying their dietary and lifestyle habits. Every lifestyle factor exerts both beneficial and harmful effects on the body. Thus, further nutritional studies assessing dietary patterns, rather than single nutrients or foods, with adjustment of systemic confounding factors on glaucoma are required.

Incorporating evidence-based lifestyle counseling into glaucoma care—within a structured framework linking systemic health to ocular pathophysiology—offers an opportunity for precision prevention. By addressing modifiable risk factors early, before irreversible RGC loss occurs, clinicians may be able to preserve visual function, extend quality-adjusted life years, and ultimately reduce the global burden of blindness.

## Figures and Tables

**Table 1:** Associations between nutrients/diet and glaucoma cited in this review article.

Nutrient/Diet	Authors, Year [Reference Number]	Study Type	Glaucoma Cases/Incidence (*n*)	Result
Carbohydrates	Hanyuda et al., 2020 [55]	Cohort study	2112	Low-carbohydrate diets were not associated with the risk of POAG. Plant-based low-carbohydrate score showed a suggestive inverse association with early paracentral visual field loss pattern.
	Moreno-Montañés et al., 2021 [56]	Cohort study	242	Higher intake of total carbohydrates is associated with a higher risk of incident glaucoma.
Proteins	Kinouchi et al., 2018 [57]	Cross-sectional	42	Higher weekly consumption of meat was inversely associated with OAG in Japanese women.
	Mylona et al., 2020 [58]	Case–control	100	Lower intake of meat was observed in OAG patients compared with controls.
	Hanyuda et al., 2022 [59]	Cohort study	1946	Higher dietary intake of BCAA was not associated with POAG risk. For the POAG subtype with early paracentral visual field loss, there was a suggestion of an inverse association in women, but not with men (P heterogeneity by sex = 0.06).
	Vergroesen et al., 2023 [60]	Cohort study	170	Adherence to the MIND diet was significantly associated with a lower incidence of OAG in contrast to adherence to the Mediterranean diet or the Dutch dietary guidelines.
Fats	Kang et al., 2004 [61]	Cohort study	474	Higher omega-3 to omega-6 ratio dietary intake increased glaucoma risk (RR 1.49).
	Pérez de Arcelus et al., 2014 [62]	Cohort study	156	Higher omega-3 to omega-6 ratio dietary intake increased glaucoma risk (HR 1.91).
	Yoserizal et al., 2019 [63]	Cross-sectional	581	Lower vegetable fat intake was associated with an increased risk of glaucoma in subjects of Japanese descent living in the Los Angeles population.
	Kai et al., 2025 [64]	Cohort study	1166	Plasma levels of omega-3 polyunsaturated fatty acids were inversely associated with glaucoma risk (HR 0.61 for a 1-unit (mmol/L) increment in plasma levels).
Vitamins	Giaconi et al., 2012 [65]	Cross-sectional	77	Higher intake of certain fruits and vegetables high in vitamins A and C and carotenoids may be associated with a decreased likelihood of glaucoma in older African American women.
	Yoserizal et al., 2019 [63]	Cross-sectional	581	Lower vitamin A was associated with an increased risk of glaucoma in subjects of Japanese descent living in the Los Angeles population.
	Kang et al., 2014 [66]	Cohort study	399	Higher total folate intake was associated with a suggestive lower risk for XFG.
	Ramdas et al., 2012 [67]	Cohort study	91	Lower intake of retinol equivalents and vitamin B1 and a high intake of magnesium were associated with an increased risk of OAG.
	Jung et al., 2018 [68]	Cross-sectional	775	Dietary nutrient intake was associated with OAG, independent of IOP. Individuals with NTG showed lower intake of niacin among nutrients.
	Kang et al., 2003 [69]	Cohort study	474	There were no associations between antioxidative properties (α-carotene, β-carotene, lutein/zeaxanthin, vitamins A, C, and E) and OAG.
	Carbone et al., 2021 [70]	Cohort study	121	Dietary vitamin D intake, supplements and serum levels are not significantly related to the risk of developing glaucoma in postmenopausal women.
	Coleman et al., 2008 [71]	Cross-sectional	95	Higher fruit/vegetable intake reduces glaucoma risk (OR 0.21 for increased fruit intake, OR 0.43 for increased vegetable intake).
	Kang et al., 2004 [72]	Cohort study	1483	Higher green leafy vegetable intake reduces glaucoma risk (MVRR 0.82).
Carotenoids	Giaconi et al., 2012 [65]	Cross-sectional	77	Higher intake of certain fruits and vegetables high in vitamins A and C and carotenoids may be associated with a decreased likelihood of glaucoma in older African American women.
	Ramdas et al., 2012 [67]	Cohort study	91	Lower intake of retinol equivalents and vitamin B1 and a high intake of magnesium were associated with an increased risk of OAG.
	Kang et al., 2003 [69]	Cohort study	474	There were no associations between antioxidative properties (α-carotene, β-carotene, lutein/zeaxanthin, vitamins A, C, and E) and OAG.
	Coleman et al., 2008 [71]	Cross-sectional	95	Higher fruit/vegetable intake reduces glaucoma risk (OR 0.21 for increased fruit intake, OR 0.43 for increased vegetable intake).
Flavonoids	Ohguro et al., 2012 [73]	Randomized controlled trial	19	The black currant anthocyanins (50 mg/d) group showed less visual field mean deviation deterioration and increased ocular blood flow during a 2-year follow-up.
	Shim et al., 2012 [74]	Case–control	332	Both anthocyanins (60 mg twice per day) and *Ginkgo biloba* extract (80 mg twice per day) were associated with improved visual function in patients with NTG.
	Ohguro et al., 2012 [75]	Interventional	21	The black currant anthocyanins (50 mg/d) group showed decreased IOP among healthy subjects at 24 months.
	Terai et al., 2014 [76]	Interventional	30	Flavonoid-rich dark chocolate intake showed a significant improvement in venous dilation 2 h after intake.
	Himori et al., 2021 [77]	Interventional	30	An 8-week oral course of antioxidant supplementation (hesperidin, crocetin, and *Tamarindus indica*) reduced plasma oxidative levels in glaucoma patients with a high oxidative stress level at baseline.
	Quaranta et al., 2003 [78]	Randomized controlled trial	27	The *Ginkgo biloba* extract (40 mg, 3 times/day) group significantly improved visual field indices without changes in IOP (4–8 weeks washout—4 weeks).
	Kang et al., 2018 [79]	Cohort study	1575	Total flavonoid intake was not associated with risk of POAG development (RR [Q5: median ~645 mg/day] versus lowest quintile [Q1: ~130 mg/day] 0.91; *p* for trend = 0.19).
Fruits, vegetables	Giaconi et al., 2012 [65]	Cross-sectional	77	Higher intake of certain fruits and vegetables high in vitamins A and C and carotenoids may be associated with a decreased likelihood of glaucoma in older African American women.
	Coleman et al., 2008 [71]	Cross-sectional	95	Higher fruit/vegetable intake reduces glaucoma risk (OR 0.21 for increased fruit intake, OR 0.43 for increased vegetable intake).
	Kang et al., 2004 [69]	Cohort study	1483	Higher green leafy vegetable intake reduces glaucoma risk (MVRR 0.82).
	Vergroesen et al., 2023 [60]	Cohort study	170	Adherence to the MIND diet was significantly associated with a lower incidence of OAG in contrast to adherence to the Mediterranean diet or the Dutch dietary guidelines.
	Mehta et al., 2023 [80]	Randomized controlled trial	1227 (intervention) vs. 1774 (control)	There was no overall benefit of dietary modification in reducing incident POAG (HR, 1.04; 95% CI, 0.96–1.12).

## Data Availability

Not applicable.

## Abbreviations

The following abbreviations are used in this manuscript:

BCAA	branched-chain amino acids
BDNF	brain-derived neurotrophic factor
CAREDS2	Carotenoids in Age-Related Eye Disease Study 2
CI	confidence interval
CNTGS	Collaborative Normal-Tension Glaucoma Study
ET-1	endothelin-1
GCL	ganglion cell layer
HR	hazard ratio
IOP	intraocular pressure
MeSH	Medical Subject Headings
MIND	Mediterranean–DASH Intervention for Neurodegenerative Delay
MPOD	macular pigment optical density
MVRR	multivariable-adjusted relative risk
NHANES	National Health and Nutrition Examination Survey
NO	nitric oxide
NTG	normal-tension glaucoma
OCT	optical coherence tomography
OCTA	optical coherence tomography angiography
OR	odds ratio
OSA	obstructive sleep apnea
POAG	primary open-angle glaucoma
RGC	retinal ganglion cell
RNFL	retinal nerve fiber layer
XFG	exfoliation glaucoma
XFS	exfoliation syndrome
